# Ocular Pathology and Genetics: Transformative Role of Artificial Intelligence (AI) in Anterior Segment Diseases

**DOI:** 10.7759/cureus.55216

**Published:** 2024-02-29

**Authors:** Priyanka Venkatapathappa, Ayesha Sultana, Vidhya K S, Romy Mansour, Venkateshappa Chikkanarayanappa, Harish Rangareddy

**Affiliations:** 1 University Health Services, St. George's University School of Medicine, St. George's, GRD; 2 Pathology, St. George's University School of Medicine, St. George's, GRD; 3 Bioinformatics, University of Visvesvaraya College of Engineering, Bangalore, IND; 4 Ophthalmology, Lebanese American University Medical Center, Beirut, LBN; 5 Biochemistry, Sri Madhusudan Sai Institute of Medical Sciences, Chikkaballapur, IND; 6 Biochemistry, Haveri Institute of Medical Sciences, Haveri, IND

**Keywords:** congenital cataract (cc), primary open angle glaucoma, cornea pathology, machine learning models, artificial intelligence in medicine

## Abstract

Artificial intelligence (AI) has become a revolutionary influence in the field of ophthalmology, providing unparalleled capabilities in data analysis and pattern recognition. This narrative review delves into the crucial role that AI plays, particularly in the context of anterior segment diseases with a genetic basis. Corneal dystrophies (CDs) exhibit significant genetic diversity, manifested by irregular substance deposition in the cornea. AI-driven diagnostic tools exhibit promising accuracy in the identification and classification of corneal diseases. Importantly, chat generative pre-trained transformer (ChatGPT)-4.0 shows significant advancement over its predecessor, ChatGPT-3.5. In the realm of glaucoma, AI significantly contributes to precise diagnostics through inventive algorithms and machine learning models, surpassing conventional methods. The incorporation of AI in predicting glaucoma progression and its role in augmenting diagnostic efficiency is readily apparent. Additionally, AI-powered models prove beneficial for early identification and risk assessment in cases of congenital cataracts, characterized by diverse inheritance patterns. Machine learning models achieving exceptional discrimination in identifying congenital cataracts underscore AI's remarkable potential. The review concludes by emphasizing the promising implications of AI in managing anterior segment diseases, spanning from early detection to the tailoring of personalized treatment strategies. These advancements signal a paradigm shift in ophthalmic care, offering optimism for enhanced patient outcomes and more streamlined healthcare delivery.

## Introduction and background

Genetics is the fundamental science that unveils the mysteries of inheritance. In ophthalmology, genetics plays a paramount role in understanding and addressing a diverse range of ocular conditions. This narrative review explores the intricate interplay between genes, heredity, and various eye disorders, shedding light on the remarkable strides made in genetic ophthalmology research, with particular emphasis on the transformative role of artificial intelligence (AI). In the world of genetics, diverse patterns of inheritance govern eye diseases, including autosomal dominant, autosomal recessive, X-linked, or mitochondrial. Understanding these patterns is crucial in the diagnosis and management of hereditary eye conditions [1].

Patterns of inheritance: In genetics, the manner in which traits or conditions are transmitted from one generation to the next is referred to as the pattern of inheritance. Various patterns dictate how genetic information is transmitted within families. Certain ocular pathologies and diseases exhibit a genetic component, meaning their potential inheritance from one's parents. These genetic eye diseases arise due to specific gene mutations or abnormalities in an individual's genetic code [1].

Autosomal dominant: Autosomal dominant inheritance refers to a pattern in which a single copy of a mutated gene on one of the non-sex chromosomes (autosomes) is sufficient to induce a disease. If one parent harbors the mutated gene, there exists a 50% probability of transmitting it to their offspring. Understanding autosomal dominant inheritance is imperative, as its presence significantly heightens the likelihood of the disease manifesting in each succeeding generation [2].

Autosomal recessive: Autosomal recessive inheritance entails the necessity of two copies of the mutated gene, one from each parent, for the manifestation of the disease. In cases where both parents are carriers (heterozygous), the disease does not manifest in them, but there is a potential for passing the mutated gene to their offspring. Understanding autosomal recessive inheritance is fundamental in discerning how a genetic disease may exhibit a pattern of skipping generations [3].

X-Linked: X-linked inheritance pertains to genes located on the X chromosome. In this pattern, genetic diseases are frequently transmitted from mothers (who possess two X chromosomes) to their sons. Daughters typically inherit one affected X chromosome from their carrier mother and one unaffected X chromosome from their father. Understanding X-linked inheritance is crucial because it predominantly affects males and may deviate from the conventional inheritance patterns seen with autosomes [4].

Mitochondrial: Mitochondrial inheritance concerns genes located in the mitochondria and small structures within cells. These genes are maternally inherited. Certain eye diseases result from mutations in mitochondrial genes. Understanding mitochondrial inheritance is essential in cases where maternal inheritance is involved [5].

The diseases with a genetic basis encompass a broad spectrum, showcasing the intricate interplay between our genetic makeup and the development of various health conditions. In the context of ophthalmology, this diverse genetic landscape unravels a fascinating tapestry of inherited eye disorders.

AI, with its remarkable capabilities in data analysis and pattern recognition, has emerged as a game-changer in the field of ophthalmology. By leveraging the power of AI for early detection, precise diagnosis, personalized treatment, and remote monitoring, we are advancing the field of ophthalmology, enhancing patient outcomes, and offering hope to those affected by challenging inherited eye conditions [6]. As AI continues to evolve, we can anticipate even greater strides in our ability to manage and ultimately discover effective treatments for ocular pathology. This narrative review explores the genetics of ocular pathology in anterior segment diseases and elucidates the pivotal role played by AI in the management of these conditions.

## Review

Corneal dystrophies (CDs)

CDs encompass a group of rare and complex hereditary disorders affecting the cornea, the clear, dome-shaped surface that covers the front of the eye. These conditions are characterized by the abnormal deposition of various substances within the cornea, including lipids and cholesterol crystals [7]. CDs are sub-classified based on the anatomic location affected: epithelial/subepithelial, epithelial-stromal, stromal, and endothelial dystrophies, according to the International Classification of Corneal Dystrophies (IC3D) [8]. As our understanding of genetics has deepened, we have come to appreciate that CDs can be attributed to specific gene mutations, and the inheritance patterns of these genes play a pivotal role in the development and progression of these disorders.

Genetic heterogeneity: One of the key features of CDs is their genetic heterogeneity. Multiple genes have been associated with different subtypes of CDs, with each gene linked to a particular form of the condition. Importantly, understanding the genetic underpinnings of CDs is instrumental in the effective diagnosis and management of these conditions [9].

Patterns of inheritance: The inheritance patterns of CDs vary depending on the specific gene involved. Some subtypes follow autosomal dominant inheritance, meaning that a mutation in a single copy of the responsible gene from either parent is sufficient for the disease to manifest [7,8]. Autosomal recessive inheritance, on the other hand, requires mutations in both copies of the gene, one from each parent. These inheritance patterns have significant implications for the likelihood of the disease appearing in each generation and the potential for carriers to transmit the condition [7,8].

Gene discoveries: CDs can exhibit a Mendelian pattern of inheritance, which may include autosomal dominant, autosomal recessive, or X-linked recessive modes. Various CDs result from genetic mutations within specific genes, such as carbohydrate sulfotransferase 6 (CHST6), keratin 3 (KRT3), keratin 12 (KRT12), phosphatidylinositol phosphate 5-kinase 3 (PIP5K3), solute carrier family 4 (sodium borate cotransporter) member 11 (SLC4A11), tumor-associated calcium signal transducer 2 (TACSTD2), transforming growth factor beta-induced (TGFBI), and UbiA prenyltransferase domain-containing protein 1 (UBIAD1) [10].

The identification of these genetic mutations has significantly enhanced our comprehension of the fundamental defects underlying these disorders. Moreover, it has facilitated the development of molecular tests for precise diagnostic purposes. While certain CDs have been linked to particular chromosomal loci, the causative genes for these conditions remain unidentified. Ongoing research is expected to uncover additional genetic insights into CDs [10].

Gene therapy for CDs: Certain genes such as visual system homeobox 1 (VSX1), dedicator of cytokinesis 9 (DOCK9), or transforming growth factor-beta (TGFB1) may have an essential, sufficient role in the disease. As our understanding of the genetic basis of CDs deepens, researchers are exploring innovative treatment approaches. Gene therapy is one promising avenue. In this approach, essential genes, such as VSX1, DOCK9, or TGFB1, can be delivered to the cornea using viral vectors or nanoparticles [11]. These gene delivery platforms are often regulated by cornea-specific promoters to ensure precise and targeted treatment.

AI-based subtyping and predictive analysis: AI-driven diagnostic tools have the potential to revolutionize the early detection of CDs. Machine learning algorithms, when provided with a wealth of clinical data and images of the cornea, can identify subtle changes that may escape the human eye. This early detection is critical for timely intervention and disease management [12]. CDs encompass a wide range of subtypes, each associated with specific genetic mutations and clinical presentations. AI algorithms not only diagnose but also precisely subtype CDs, enabling tailored treatments to each patient's conditions [13].

In a study conducted by Gu et al., a groundbreaking approach to the identification of corneal diseases was demonstrated through the implementation of a novel deep-learning algorithm. The devised hierarchical deep learning network comprises a family of multi-task, multi-label learning classifiers, each representing specific levels of eye diseases defined by a pre-established hierarchical eye disease taxonomy [14].

The algorithm was meticulously trained end-to-end, utilizing a dataset of 5,325 ocular surface images extracted from a retrospective dataset. Subsequently, its performance was rigorously assessed against the expertise of 10 ophthalmologists using a prospective clinic-based dataset. This dataset comprised 510 outpatients newly enrolled with various corneal diseases, including infectious keratitis, non-infectious keratitis, CD or degeneration, and corneal neoplasm. The algorithm achieved an area under the receiver operating characteristic (ROC) curve, exceeding 0.910 for each corneal disease type. The algorithm demonstrated sensitivity and specificity comparable to, or even surpassing, the average values exhibited by all participating ophthalmologists. Confusion matrices revealed noteworthy similarities in misclassification patterns between the algorithm and human experts. Furthermore, the algorithm outperformed all four previously reported methods in the identification of corneal diseases [14]. A unique feature of the study is the introduction of a multi-level eye disease-guided loss function, specifically tailored to capture the fine-grained variability of eye disease features. This innovation contributes to the algorithm's ability to discern subtle nuances within the spectrum of corneal diseases. This suggests its potential as a valuable tool for computer-assisted corneal disease diagnosis, showcasing superior performance in comparison to existing methodologies.

Within the realm of AI tools, chat generative pre-trained transformer (ChatGPT), an advanced large language model (LLM) engineered by OpenAI (San Francisco, CA), has garnered recent interest and is poised with significant promise for grasping clinical knowledge and providing pertinent information. Utilizing deep learning methodologies, ChatGPT proficiently produces coherent and contextually relevant text in response to user inputs [15].

In a study by Delsoz et al., the diagnostic capabilities of ChatGPT-4.0 and ChatGPT-3.5 for corneal eye diseases were evaluated and compared with human experts. Twenty cases, spanning corneal infections, dystrophies, degenerations, and injuries, were randomly selected from a publicly accessible online database. Case descriptions were input into both ChatGPT-4.0 and ChatGPT-3.5 for provisional diagnoses, and results were compared with those of three cornea specialists. ChatGPT-4.0 demonstrated a notable diagnostic accuracy of 85%, outperforming ChatGPT-3.5, which achieved 60%. Human experts achieved accuracies ranging from 90% to 100%. Inter-observer agreements showed that ChatGPT-4.0 exhibited higher consistency (65%) with ChatGPT-3.5, while agreements with human experts ranged from 75% to 85%. The study concludes that ChatGPT-4.0 shows marked improvement over ChatGPT-3.5 in diagnosing corneal conditions, indicating promising potential for clinical integration [16].

**Figure 1:**
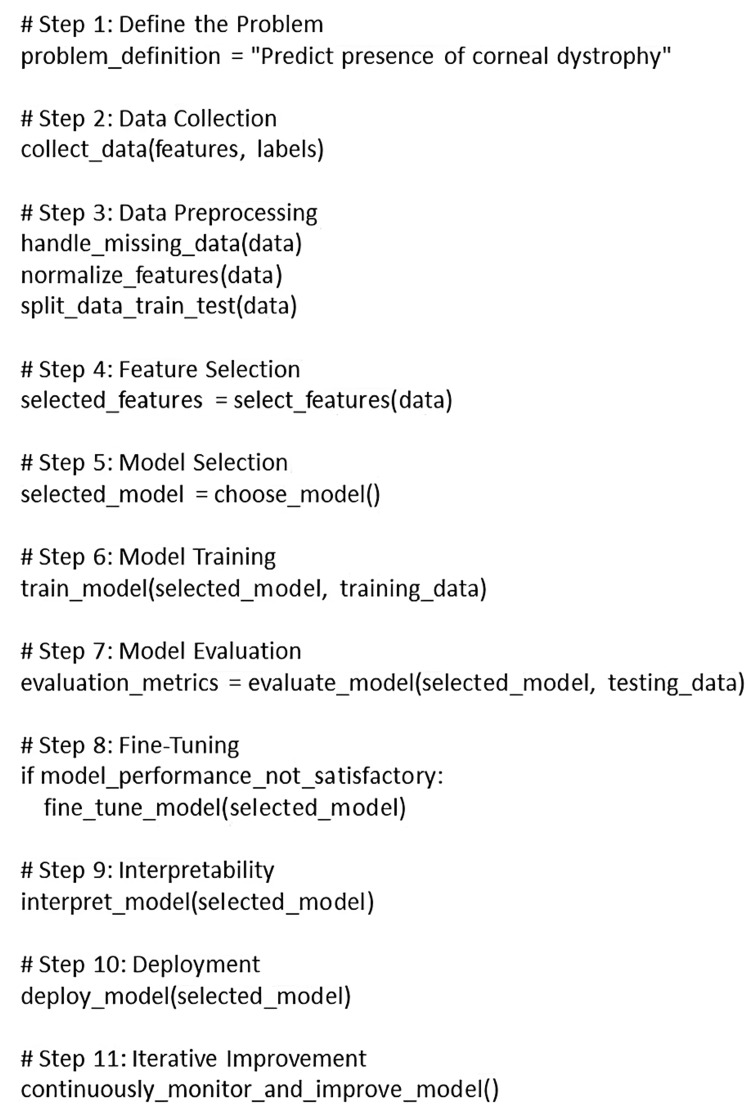
Basic structure of a machine learning algorithm for predicting corneal dystrophy The figure has been created by Vidhya K S.

By analyzing historical patient data, including genetic information, clinical findings, and treatment responses, AI can provide prognostic insights. This predictive analysis assists in creating personalized treatment plans and establishing realistic expectations for patients. AI's capacity to process extensive amounts of medical literature and clinical data helps ophthalmologists make well-informed decisions about the most suitable treatment strategies for individuals with CD. Its potential extends to guiding the selection of the optimal course of action, be it gene therapy, surgical intervention, or conservative management. An example of a machine learning algorithm for CD prediction is depicted below in the form of a simple basic structure, as shown in Figure 1.

Glaucoma

Glaucoma is a heterogeneous group of eye diseases characterized by progressive damage to the optic nerve, which can ultimately lead to vision loss and blindness. While various factors contribute to the development of glaucoma, such as elevated intraocular pressure, there is a growing awareness of the substantial impact of genetics on an individual's susceptibility to this condition [17].

Genetic complexity: Glaucoma exhibits a complex genetic landscape. It is not caused by a single gene, but rather, it involves the interplay of multiple genes, each contributing to an individual's overall risk of developing the disease. Several genes have been associated with different subtypes of glaucoma, making it a multifactorial condition [18].

Myocilin and cytochrome P450 1B1 (CYP1B1): Myocilin was the first gene identified to be linked to glaucoma. Mutations in the myocilin gene are implicated in open-angle glaucoma, a prevalent form of the disease. Located on chromosome 1, this gene encodes a protein secreted within the trabecular meshwork, the drainage angle of the eye. Mutations in the myocilin gene can lead to damage in the trabecular meshwork, thereby hindering the aqueous outflow and contributing to the onset of glaucoma [19].

CYP1B1 emerges as another gene associated with congenital glaucoma. In some cases, individuals may carry dual mutations, with one in the myocilin gene and another in the CYP1B1 gene, leading to a more aggressive and earlier-onset form of glaucoma. In the context of primary congenital glaucoma, genetic studies employing linkage analysis have identified CYP1B1 as a significant genetic locus situated on chromosome 2p22.2. This form of glaucoma is associated with an autosomal recessive inheritance pattern and sporadic occurrences. Notably, CYP1B1 is implicated in steroid metabolism and plays a crucial role in the development of the anterior segment of the eye [20]. Through genetic linkage analysis, latent transforming growth factor beta binding protein 2 (LTBP2) emerges as a notable genetic locus located on chromosome 14q24.3. This form of glaucoma exhibits an autosomal recessive inheritance pattern. LTBP2, responsible for encoding an extracellular matrix protein, plays a crucial role in cell adhesion, elastic fiber assembly, and microfibril structure [21]. In the realm of whole exome sequencing, two additional genes associated with primary congenital glaucoma have been identified - TEK receptor tyrosine kinase/tyrosine kinase with immunoglobulin-like and EGF-like domains 2 (TEK/TIE2) on 9p21.2 and angiopoietin 1 (ANGPT1) on 8q23.1. Both genes exhibit an autosomal dominant inheritance pattern. TEK/TIE2 encodes a receptor tyrosine kinase and plays a pivotal role in the development of the aqueous outflow pathway. Concurrently, ANGPT1, serving as a ligand for TEK, also contributes to the development of the aqueous outflow pathway [22,23]. The identification of these genes through whole exome sequencing enhances our understanding of the genetic landscape underlying primary congenital glaucoma, providing valuable insights into the molecular mechanisms that govern the condition and potential avenues for targeted therapeutic interventions.

Complex inheritance patterns: Glaucoma manifests with various inheritance patterns. Some cases are sporadic or arise as a result of complex inheritance, where multiple genes and environmental factors are involved. However, there are instances of familial glaucoma, where the condition is passed down from generation to generation. The inheritance pattern varies by subtype, with some forms showing autosomal dominant, autosomal recessive, or X-linked patterns. Understanding the inheritance pattern is essential for genetic counseling and predicting the likelihood of the condition affecting family members [18].

In the evolution of glaucoma diagnostics, the integration of image processing and machine learning has emerged as a transformative force. Originating in the 1950s with early image processing applications, the incorporation of machine learning into glaucoma diagnosis arose in the 1990s, particularly through the application of neural networks to visual fields (VFs) [24]. Subsequent neural network models, analyzing VFs characterized by threshold sensitivity or total deviations, showcased their efficacy in glaucoma diagnosis [25,26]. Comparative studies explored machine learning models, such as multilayer perceptron (MLP), support vector machine (SVM), and ensemble learning, revealing superior accuracy compared to traditional VF indexes. Despite the limitations of raw VFs for deep convolutional neural network (CNN) analysis, some researchers employed deep CNN models on VF printouts, achieving substantial success in differentiating normal from glaucomatous VFs [27].

Asaoka et al. introduced a random forests (RF) machine learning method designed to differentiate VFs in preperimetric open-angle glaucoma eyes. The success of this method, evidenced by favorable results in the area under the ROC curve and significant total deviation differences, marked a pivotal development [28]. Another notable contribution came from Li et al., who developed a CNN system employing the vision geometrical group (VGG) network structure. This CNN, pre-trained on the ImageNet dataset and adapted for glaucoma detection, outperformed rule-based methods and non-deep machine learning algorithms such as RF. With an accuracy of 0.876, specificity of 0.826, and sensitivity of 0.932, the CNN demonstrated superior glaucomatous VF discrimination compared to various other AI VF algorithms [29]. This underscores the potential of advanced AI techniques in enhancing glaucoma diagnostics.

Dixit et al. performed a retrospective analysis aimed to enhance the detection of glaucoma progression by evaluating a convolutional long short-term memory (LSTM) neural network using a longitudinal dataset that combines VF and clinical information. The study included individuals with four or more VF results and corresponding baseline clinical data from an initial dataset of 672,123 VF results and 350,437 samples of clinical data. After applying exclusion criteria to ensure data reliability, 11,242 eyes remained. Glaucoma progression was determined using three common algorithms (VF index slope, mean deviation slope, and point-wise linear regression). Two machine learning models, one trained exclusively on VF data and another on both VF and clinical data, were tested. The convolutional LSTM network demonstrated 91%-93% accuracy with different glaucoma progression algorithms given four consecutive VF results. The model trained on both VF and clinical data (AUC: 0.89-0.93) outperformed the model exclusively trained on VF results (AUC: 0.79-0.82; P < 0.001) [30]. This study suggests that the convolutional LSTM architecture, adept at capturing spatiotemporal features in VFs, improves the assessment of glaucoma progression, with the inclusion of clinical data enhancing diagnostic ability, aligning with clinical practices in glaucoma management.

Li et al. conducted a study evaluating the performance of 'iGlaucoma,' a deep learning system (DLS) embedded in a smartphone application, for detecting glaucomatous VF changes. The study included 1,614,808 data points from 10,784 VFs (5,542 patients) across two phases in seven centers in China. In Phase I, 1,581,060 data points from 10,135 VFs of 5,105 patients were used to train, validate, and test the DLS. The diagnostic performance of the DLS was compared to six ophthalmologists using three independent test sets. The DLS outperformed all six ophthalmologists in Phase I, achieving an AUC of 0.834-0.877, sensitivity of 0.831-0.922, and specificity of 0.676-0.709. In Phase II, iGlaucoma demonstrated 0.99 accuracies in recognizing different patterns in the pattern deviation probability plots region, with corresponding AUC, sensitivity, and specificity of 0.966 (0.953-0.979), 0.954 (0.930-0.977), and 0.873 (0.838-0.908), respectively. The study highlights the superior performance of the DLS compared to ophthalmologists in detecting glaucoma, emphasizing the potential of smartphone-based applications for efficient glaucoma assessment [31].

AI excels in precisely evaluating the optic nerve, a critical component in glaucoma diagnosis. AI systems can analyze optical coherence tomography (OCT) scans and detect structural changes in the optic nerve head and retinal nerve fiber layer (RNFL), providing vital information for clinicians. A three-dimensional (3D) deep learning algorithm is integrated with spectral-domain OCT (SD-OCT) to assess degeneration in the optic nerve head of glaucomatous eyes. The initial 3D DLS, developed by Ran et al., employed ResNet structures for analyzing glaucomatous lesions in optic nerve head volume data obtained through SD-OCT. In comparison to 2D models, this system leverages 3D deep learning algorithms, yielding improved outcomes. Its diagnostic accuracy is further heightened by the precise segmentation of retinal layers and quantification of the RNFL and macular ganglion cell complex [32]. Subsequently, Noury et al. employed a 3D convolutional neural network (CNN) trained to differentiate glaucoma using datasets from Stanford University, Hong Kong, India, and Nepalese sources. The model demonstrated effective discrimination across OCT images from diverse ethnic groups, with a specific focus on the lamina cribrosa region for glaucoma diagnosis. This approach aligns with clinical parameters such as cup diameter/volume or rim area/volume [33].

**Figure 2:**
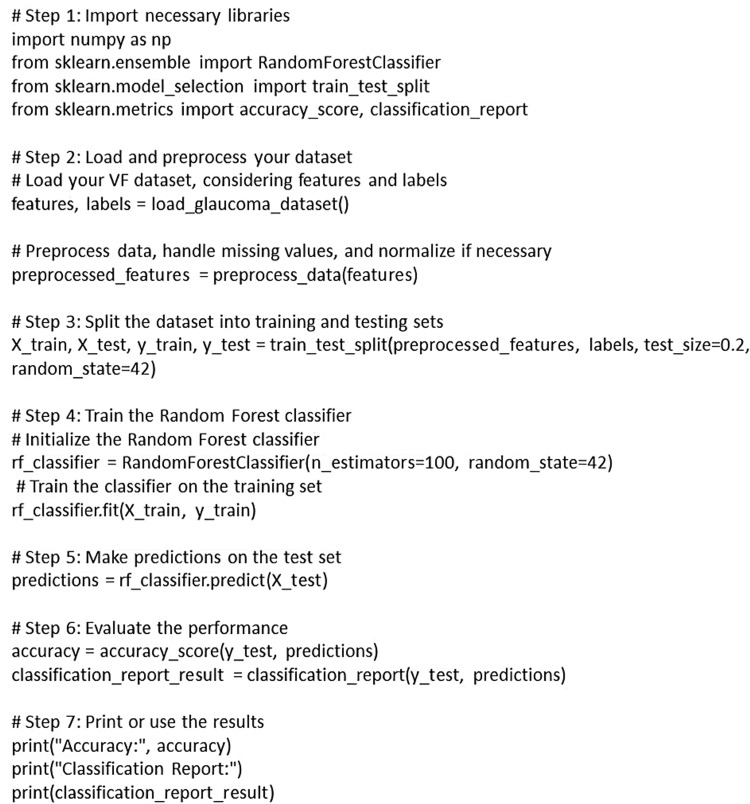
Steps for the application of random forest for differentiating visual fields in glaucoma The figure has been created by Vidhya K S.

AI is adept at tracking disease progression. By analyzing a patient's longitudinal data, including VF tests and imaging results, AI can detect subtle changes that might not be apparent to the human eye. This enables ophthalmologists to make informed decisions on when to adjust treatment strategies. Recent advancements have introduced two innovative deep learning approaches, employing a CNN and a recurrent neural network (RNN), for the anticipation of future VF outcomes. In a study by Eslami et al., the precision of the previously introduced CNN and RNN models in forecasting VF alterations over time was investigated. Despite exhibiting some efficacy, both models displayed inaccuracies when predicting VF changes in patients with severe glaucoma. Notably, they tended to significantly underestimate the extent of VF loss in such patients and exhibited suboptimal performance in cases with substantial VF changes at baseline and follow-up [34]. These limitations could potentially impact their clinical utility. To address these challenges, ongoing exploration involves variational autoencoder deep learning models, with the aim of mitigating these issues to a certain extent [35]. An example of the application of RF in glaucoma VF differentiation is provided in the form of a basic structure below in Figure 2.

Congenital cataract (CC)

CC refers to the clouding or opacification of the lens of the eye that is either present at birth or develops during early childhood. This condition has a substantial genetic component, and understanding the genetic factors involved is essential for accurate diagnosis, risk assessment, and the development of effective treatment strategies [36].

Genetic complexity: Cataracts, a common cause of vision impairment, exhibit diverse inheritance patterns. In autosomal dominant inheritance, a single mutated gene copy from one parent suffices to trigger the condition, with genes such as α-crystallin (CRYAA and CRYAB) and γ-crystallin (CRYGC and CRYGD) implicated [37,38]. In autosomal recessive cataracts, two copies of the mutated gene, one from each parent, are required for manifestation. An example is the elongation of very long chain fatty acids protein 4 (ELOVL4) gene. Multiple genes are associated with autosomal recessive CC [39]. In X-linked inheritance, the mutated gene is located on the X chromosome. Mutations in genes such as the Nance-Horan syndrome (NHS) gene can lead to X-linked CC. This pattern of inheritance primarily affects males and is passed from carrier females [40]. Conversely, sporadic cases and de novo mutations arise independently, not through inheritance, with environmental factors like prenatal toxin exposure contributing to such occurrences [41]. This diversity underscores the intricate interplay between genetic and environmental factors in CC etiology, highlighting the complexity of its origins.

While the majority of Mendelian isolated cataracts are typically identified within the initial year of life, some cases may manifest as late as early adulthood. Currently, there are approximately 71 mapped loci associated with cataracts, revealing 56 identified causative genes [42]. Beyond isolated instances, inherited cataracts can be integral components of multi-systemic diseases [43]. Furthermore, certain mutations linked to cataracts can induce extra lenticular effects as part of a broader developmental sequence. A notable illustration of this phenomenon involves crystallin mutations, which can lead to profound early disruptions in lens development, subsequently influencing the development of the anterior chamber and, in extreme cases, resulting in microcornea or microphthalmia. Lastly, the onset of severe cataracts in early stages can impede visual input during the critical developmental period, potentially obstructing the formation of neural pathways connecting the retina to the optic cortex, consequently causing conditions such as nystagmus or even blindness [42].

Harnessing the capabilities of AI holds promising implications for various stages of CC management. In early detection and diagnosis, AI-driven image analysis offers a valuable tool for scrutinizing retinal images, enabling the identification of cataract indicators in newborns and facilitating prompt intervention. The application of AI extends to risk assessment, utilizing genetic data and family history analysis to predict the likelihood of CCs in infants. This proactive approach enables continuous monitoring and early intervention for at-risk individuals. Additionally, AI contributes to surgical assistance by providing real-time guidance during cataract surgery, aiding surgeons in critical aspects, such as lens selection, incision planning, and intraoperative decision-making, thereby enhancing the precision of surgical procedures [44].

In a case-control study conducted by Lin et al. at the Zhongshan Ophthalmic Center, a practical machine-learning model for CC identification was developed. The study involved 2005 subjects, encompassing 1,274 children with CCs and 731 healthy controls. Utilizing birth conditions, family medical history, and family environmental factors, CC identification models were established employing the RF and adaptive boosting methods. Trained on 1,129 CC cases and 609 healthy controls, these models underwent internal fourfold cross-validation and external validation involving 145 CC cases and 122 healthy controls. Robust performance was demonstrated across various scenarios, including four datasets with progressively reduced proportions of CC patients, mimicking a clinical environment with lower disease prevalence. The CC identification models exhibited notable discrimination, with an AUC of 0.91 (95% confidence interval: 0.88-0.94) in bilateral cases and 0.82 (0.77-0.89) in unilateral cases during fourfold cross-validation. External validation further affirmed their efficacy, yielding AUCs of 0.93±0.05 in bilateral cases and 0.86±0.01 in unilateral cases. The clinical tests demonstrated stable performance, recording AUCs ranging from 0.94 to 0.96 across four subgroups as determined by RF. Moreover, a family history of CC, lower parental education levels, and the presence of comorbidities have been identified as the top three most pertinent factors for the diagnoses of both bilateral and unilateral CC [45]. This study underscores the potential of machine learning models in efficiently identifying CC, providing valuable insights for clinical applications, as it holds promise as an additional screening method.

**Figure 3:**
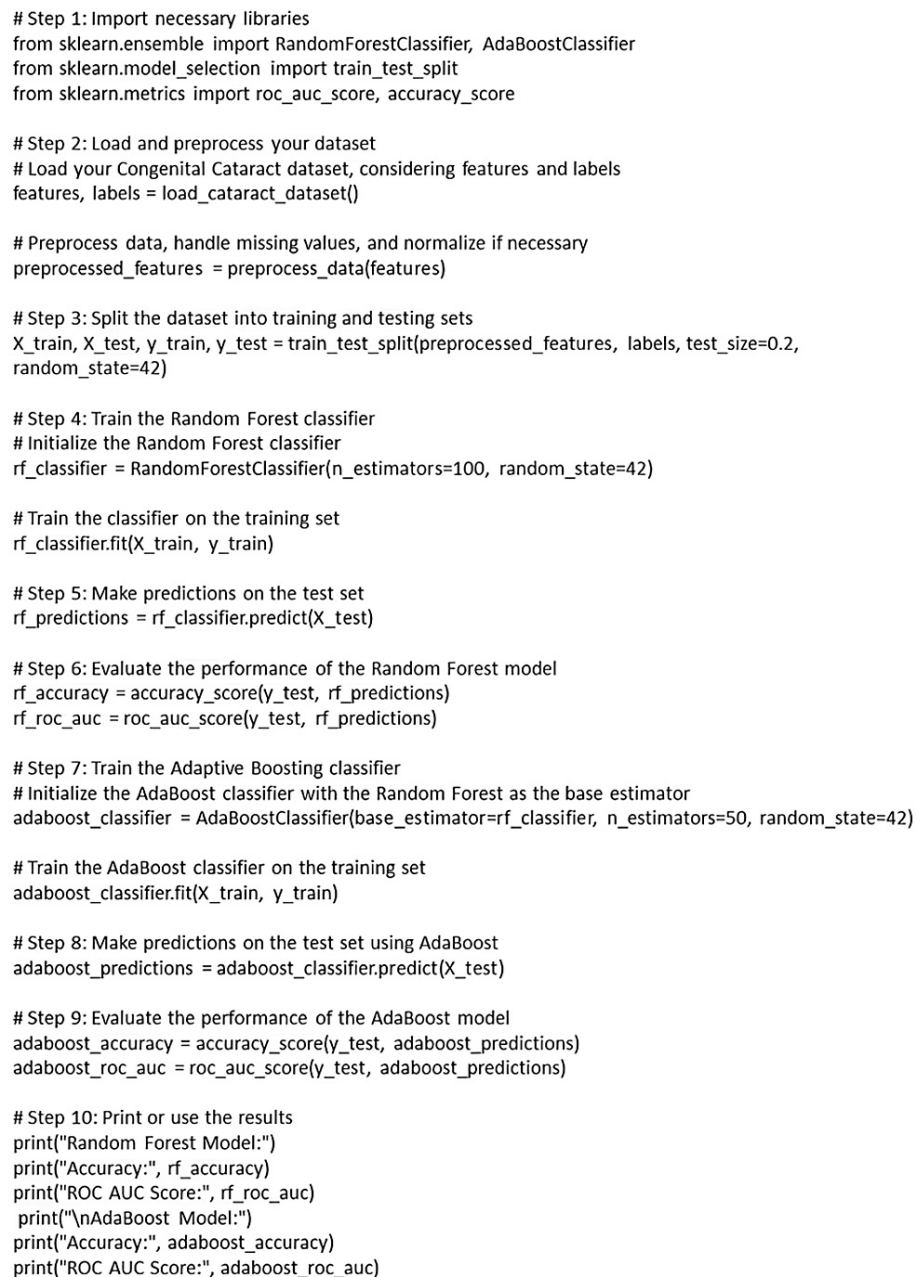
Basic structure of the congenital cataract identification model The figure has been created by Vidhya K S.

Long et al. developed CC-Guardian, an AI tool utilizing Bayesian and deep learning algorithms, as a tool to help liberate patients from the burden of prolonged monitoring and periodic visits of chronic diseases such as CC. This AI tool incorporated individualized prediction, scheduling, and telehealth follow-up computing to enhance efficiency and accuracy. Through internal validation and validation across multiple resources, CC-Guardian demonstrated high sensitivity and specificity. The integration of the AI tool with a web-based smartphone app facilitated the creation of a prototype prediction-telehealth cloud platform, forming a comprehensive intelligent follow-up system. A retrospective self-controlled test highlighted the system's ability to detect and address complications at earlier stages, showcasing its effectiveness compared to conventional methods. Additionally, the intelligent follow-up system demonstrated a reduction in socioeconomic burdens, emphasizing its potential as a valuable tool in healthcare management [46]. A basic structure of a CC identification model is suggested in Figure 3.

## Conclusions

Genetics has emerged as a pivotal player in the field of ophthalmology. Our expanding knowledge of the genetic basis of eye diseases has opened up new possibilities for diagnosis, treatment, and even prevention. From understanding the inheritance patterns of eye disorders to exploring gene therapies and innovative approaches such as stem cell research, the future of ophthalmology is intertwined with genetics. As we delve deeper into the genetic foundations of eye diseases, we gain insights that pave the way for personalized treatments and the preservation of vision. In this ever-evolving field, the capacity of AI to analyze extensive amounts of medical literature and clinical data helps ophthalmologists make informed decisions regarding the most suitable treatment strategies for patients with ocular pathologies. It assists in determining the optimal course of action, be it gene therapy, surgical intervention, or conservative management. In an era where telemedicine is gaining prominence, AI-enabled teleophthalmology platforms allow patients to receive remote assessments and monitoring. This is particularly advantageous for individuals with ocular pathology, as it can allow real-time tracking of their conditions without the need for frequent visits to healthcare facilities.

AI systems demonstrate remarkable precision in analyzing ocular images, including microscopy and OCT scans. Their ability to detect subtle changes aids in treatment decisions and monitoring of disease progression. By efficiently sifting through extensive datasets, AI facilitates data-driven research, identifying correlations, potential risk factors, and treatment outcomes. This research not only advances our understanding of ocular diseases but also paves the way for the development of novel therapies. The integration of AI in clinical practice streamlines processes, reducing diagnostic delays and enhancing the overall patient experience. Patients with ocular pathology can benefit from quicker, more accurate diagnoses, and individualized treatment plans. In conclusion, AI plays an integral role in ophthalmology, unraveling the complexities of inherited eye diseases and offering hope through gene therapy, and targeted treatments. The field continues to advance, with ongoing research expanding our understanding and providing new avenues for managing these conditions.
